# Smoking is Associated with Poorer Quality-Based Outcomes in Patients Hospitalized with Spinal Disease

**DOI:** 10.3389/fsurg.2015.00020

**Published:** 2015-05-28

**Authors:** Erica F. Bisson, Christian A. Bowers, Samuel F. Hohmann, Meic H. Schmidt

**Affiliations:** ^1^Department of Neurosurgery, Clinical Neurosciences Center, University of Utah, Salt Lake City, UT, USA; ^2^Comparative Data and Informatics Research, University HealthSystem Consortium, Chicago, IL, USA

**Keywords:** smoking, spine, surgery, healthcare costs, value, modifiable risk factors, patient outcomes, quality improvement

## Abstract

**Study design:**

Retrospective cross-sectional database analysis.

**Objective:**

The cost of spine surgery is growing exponentially, and cost-effectiveness is a critical consideration. Smoking has been shown to increase hospital costs in general surgery, but this impact has not been reported in patients with spinal disease. The objective of this work was to evaluate the effect of smoking on cost and complications in a large sample of patients admitted for treatment of spinal disease.

**Methods:**

In 2012, the authors identified all inpatient admissions to all University HealthSystem Consortium (UHC) hospitals from 2005 to 2011 for spinal disease based on the principal diagnosis ICD-9-CM codes from the prospectively collected UHC database. Patient outcomes – including length of stay; complication, readmission, intensive care unit admission rates; and total cost – were compared for non-obese smokers and non-smokers using a two-sample *t*-test.

**Results:**

There were 137,537 patients, including 136,511 (122,608 non-smokers and 13,903 smokers) in the 4 largest diagnostic groups. Smoking was associated with increased complications and worse outcomes in three of these four groups. All outcomes in the two largest groups – fracture and dorsopathy – were worse in the smoking patients.

**Conclusion:**

Smoking patients admitted for spinal disease in the sample had worse outcomes, increased complications, and higher costs than their non-smoking counterparts. In the current health-care climate focused on cost-effectiveness, smoking represents a potentially modifiable area for cost reduction.

## Introduction

Approximately 1.2 million spinal surgeries are performed annually in the United States for traumatic, oncologic, degenerative, and other conditions (1, 2). Fusion surgeries alone cost more than $10 billion, and the overall cost of spine care approaches $100 billion, accounting for nearly 10% of the nation’s total healthcare expenditures (1, 2). While the annual increase in the cost of healthcare in general has been at its lowest recorded rates of the last 50 years, at 3.8 and 3.9%, respectively, for 2009 and 2010, spine care expenditures as recently as 2005 were estimated to have increased 65% from their 1997 levels (3). The reason for this exponential increase remains unclear.

The shift over the last few decades to the use of more instrumentation in spinal surgery is well-documented, with increasing numbers of both instrumented levels per spinal procedure and spinal fusions altogether (4). Some have argued that the cost of instrumentation has played a large role in the cost increase seen for spinal surgery in the last decade, but there are likely to be other potentially modifiable variables that affect the overall cost of hospitalization for a patient with spinal disease. Recently, there has been a call by the Institutes of Medicine (5) for research on cost-effectiveness in many fields of medicine, including spine surgery, to help define and improve value in medical care. To address this call, the medical community will need not only to identify the optimal treatment strategies based on cost-effective studies but also to understand patient and hospital variables that may impact the overall cost, both monetary and non-monetary, of these procedures.

A recent review summarized the results from multiple studies documenting the negative impact of smoking on the overall hospital care of surgical patients, including increased risk of perioperative complications, morbidity, mortality, and admission to the intensive care unit (ICU) in patients undergoing orthopedic, general surgery, plastic surgery, and cardiac procedures (6–10). The complications associated with smoking include wound infections, delayed wound healing, pneumonia, and myocardial infarction (9). Even pediatric surgery is affected, as pediatric patients with more secondhand smoking exposure have been shown to have higher rates of anesthesia-associated respiratory complications (9). In addition to its impact on the medical care of patients, smoking negatively affects costs associated with surgery. In 2012, Kamath et al. (10) reported that among veterans undergoing general surgical procedures, those who used tobacco had significantly higher hospital-associated costs. With approximately 10 million surgical procedures currently done on smokers in the United States annually, including spinal procedures, it is imperative that the contribution of this potentially modifiable risk to the overall hospital cost of these procedures is defined (10).

As payment for the delivery of healthcare is changing, quality improvement and cost reduction in medical care are increasing on the agenda for all healthcare institutions. The goal of this study was to evaluate the association of smoking with cost and complications in a large sample of patients admitted for spinal disease at both academic and non-academic institutions using the prospectively collected data in the University HealthSystem Consortium (UHC) database. Although the use of an administrative database limits the ability to evaluate individual patient-level data, it nevertheless provides the opportunity for a preliminary analysis that may be the basis for future in-depth investigation.

## Materials and Methods

### UHC database

The UHC is a network of 116 academic medical centers and 276 of their affiliated hospitals, comprising >90% of non-profit academic medical centers in the country. The UHC database contains demographic, financial, administrative, diagnostic, and procedural information in addition to specific hospital stay information such as medication use, ICU admission rates/length of stay (LOS), total hospital LOS, morbidity rates, mortality rates, complications, and readmission rates (11, 12). The UHC database provides the ICD-9-CM code for almost every hospital discharge, and although it was originally used for improving cost-effectiveness, quality healthcare, safety, and overall outcomes through comparing the various member institutions, multiple groups in other specialties, especially general surgery, have used this database to identify specific variables that impact outcomes (11–15). The patient data set was de-identified before data gathering and therefore is exempt from institutional review board approval.

### Patient population

In 2012, the authors queried the UHC database in a retrospective cross-sectional analysis of all UHC hospitals from 2005 to 2011 to identify all inpatient admissions for treatment of spinal disease based on the principal diagnosis ICD-9-CM codes, including cord injury, congenital, curvature, dislocation, dorsopathy, which is defined as “a condition in which there is a deviation from or interruption of the normal structure or function of the spine,” fracture, and sprains/strains. Obese patients (defined either by a primary ICD-9-CM code of “obesity” or “morbid obesity” or by a body mass index >25) were excluded because obesity is a known independent risk factor for adverse outcomes and is associated with increased cost, more complications, and higher infection rates in spine surgery patients (16–19). The remaining non-obese patients with each UHC database diagnosis code were then further categorized as smokers or non-smokers. The primary aim of this study was to evaluate whether smoking has an association with either cost or quality-based outcomes in specific cohorts of patients admitted with spinal disease as their principal diagnosis.

### Outcomes

Primary outcomes were hospital LOS, ICU admission, 7-, 14-, and 30-day readmission rates, complication rates, and total direct cost associated with the hospital admission. Complications were defined by ICD-9-CM code and included a list of 39 potential surgical complications, such as wound infection, myocardial infarction, and urinary tract infection (see Appendix).

### Statistical analysis

The authors compared the outcomes for all non-obese spine patients that were non-smokers with outcomes for smokers using a two-sample *t*-test with a 95% confidence interval. Pooled variance was appropriate for almost all of the comparisons. Statistical analysis was performed using SAS, Enterprise Guide Version 4.3.

## Results

There were a total of 137,537 non-obese patients admitted with spinal disease during the study period among all pathology groups, including 136,511 total patients among the dorsopathy, fracture, curvature, and congenital groups, the four largest cohorts. Approximately 10% of these were smokers (13,903) and almost 90% were non-smokers (122,608). The non-smoking patients included 92,553 dorsopathy patients, 16,584 fracture patients, 1,962 congenital patients, and 11,509 curvature patients, whereas the smoking patients included 11,384 dorsopathy patients, 1,052 fracture patients, 228 congenital patients, and 1,239 curvature patients.

Statistical analysis demonstrated that smoking was associated with increased complications and worse outcomes in three of the four largest diagnosis groups. Smokers in the dorsopathy group had worse outcomes across all measured variables when compared with non-smokers (Table 1), with a mean LOS that was 1.01 days longer (*p* < 0.001), a 6% higher ICU admission rate (*p* < 0.001), a complication rate that was 1.73% higher (*p* < 0.001), and a 0.76% higher 7-day readmission rate (*p* = 0.00), a 1.56% higher 14-day readmission rate (*p* < 0.001), and a 2.08% higher 30-day readmission rate (*p* < 0.001). Smoking patients in the fracture group also had significantly worse outcomes across all measured outcomes (Table 2), with a 4.76-day longer mean LOS (*p* < 0.001), a 13% higher ICU admission rate (*p* < 0.001), a 7.12% higher complication rate (*p* < 0.001), and a 1.38% higher 7-day readmission rate (*p* < 0.01), a 2.25% higher 14-day readmission rate (*p* < 0.01), and a 3.75% higher 30-day readmission rate (*p* < 0.001). The smokers in the congenital group had worse LOS (0.57 days longer, *p* = 0.04) and higher 30-day readmission rates (3.54% higher, *p* = 0.02), but there was not a significant difference in the other measured outcomes of ICU admission, complication rate, and 7- and 14-day readmission rates (Table 3). Finally, the smokers in the curvature group had worse 14-day (1.88% higher, *p* < 0.01) and 30-day (3.04% higher, *p* < 0.001) readmission rates (Table 4), but there was not a significant difference in the other measured outcomes.

**Table 1:** **Outcomes of patients admitted for spinal treatment for dorsopathy**.

	Smoker	Non-smoker	*p*-Value
Mean LOS (days)	4.87	3.77	0.00
Mean ICU admission rate (%)	18	12	0.00
Mean complication rate (%)	6.97	5.24	0.00
Mean 7-day readmission rate (%)	2.80	2.04	0.00
Mean 14-day readmission rate (%)	4.84	3.28	0.00
Mean 30-day readmission rate (%)	6.89	4.81	0.00
Mean total cost ($)	23,185	18,889	0.00

*LOS, length of stay; ICU, intensive care unit*.

**Table 2:** **Outcomes of patients admitted for spinal treatment for fracture**.

	Smoker	Non-smoker	*p*-Value
Mean LOS (days)	19.13	14.37	0.00
Mean ICU admission rate (%)	71	58	0.00
Mean complication rate (%)	26.90	19.78	0.00
Mean 7-day readmission rate (%)	3.98	2.60	0.00
Mean 14-day readmission rate (%)	7.03	4.83	0.00
Mean 30-day readmission rate (%)	11.22	7.47	0.00
Mean total cost ($)	62,039	45,699	0.00

*LOS, length of stay; ICU, intensive care unit*.

**Table 3:** **Outcomes of patients admitted for spinal treatment for congenital disease**.

	Smoker	Non-smoker	*p*-Value
Mean LOS (days)	4.91	4.34	0.04
Mean ICU admission rate (%)	14	10	0.09
Mean complication rate (%)	5.26	6.47	0.43
Mean 7-day readmission rate (%)	3.51	2.09	0.17
Mean 14-day readmission rate (%)	4.82	3.47	0.29
Mean 30-day readmission rate (%)	8.33	4.79	0.02
Mean total cost ($)	28,889	24,642	0.01

*LOS, length of stay; ICU, intensive care unit*.

**Table 4:** **Outcomes of patients admitted for spinal treatment for curvature**.

	Smoker	Non-smoker	*p*-Value
Mean LOS (days)	6.89	6.78	0.63
Mean ICU admission rate (%)	35	33	0.47
Mean complication rate (%)	12.35	11.94	0.68
Mean 7-day readmission rate (%)	3.13	2.46	0.15
Mean 14-day readmission rate (%)	6.46	4.58	0.00
Mean 30-day readmission rate (%)	10.17	7.13	0.00
Mean total cost ($)	40,674	39,850	0.51

*LOS, length of stay; ICU, intensive care unit*.

Total hospital costs were significantly higher in smokers with dorsopathy, congenital, and fracture as primary diagnosis codes (Tables 1–3). The total hospital cost for patients with dorsopathy was $23,185 for smokers vs. $18,889 for non-smokers (*p* < 0.001). Costs for smokers with fractures were $62,039 while those for non-smokers with fractures were $45,699 (*p* < 0.001), and the total direct cost for smokers treated for congenital causes was $28,889 compared with $24,642 for non-smokers (*p* = 0.01).

## Discussion

Despite abundant evidence documenting the negative impact of smoking on surgical outcomes and cost, the literature documenting the negative association in patients with spinal disease is scant. The authors present evidence of increased costs associated with smoking in patients admitted for treatment of spinal disease. Smoking in the dorsopathy and fracture groups was associated with worse outcomes across all the study variables of LOS, ICU admission, 7-, 14-, and 30-day readmission rates, complication rates, and, ultimately, total direct cost. Patients in the congenital group who smoked had also significantly longer LOS, increased total direct cost, and higher 30-day readmission rates. Smokers in the curvature group had higher 14- and 30-day readmission rates when compared with non-smokers. The total direct cost of care was also much higher in smoking patients, and the difference was statistically significant for patients with dorsopathy, fracture, and congenital disease with a difference of $4,296, $16,370, and $4,247, respectively. In fracture patients, this increase in total cost was more than 25% of the overall direct cost of the hospitalization.

Although an avalanche of research has documented the harmful health effects of cigarette smoking, it remains the leading preventable cause of morbidity and mortality in the U.S. and accounts for 6–8% of national health-care costs (10, 20, 21). With an estimated 30% smoking incidence among patients undergoing elective general surgery, over 10 million surgical procedures are performed on smokers annually in the U.S. (10). Smoking in surgical patients has been shown to be associated with increased complication rates including increased mortality, wound infection, reoperation rates, ICU stay, overall LOS, 30-day mortality, 1-year mortality, and numerous other negative pulmonary, cardiovascular, and wound healing effects (6, 8–10, 18, 20, 22–25), all of which are associated with increased healthcare costs (10, 26–28).

The socioeconomic burden of spinal care, both operative and non-operative, on healthcare costs is substantial (1). Smoking has been shown repeatedly to lead to worse fusion rates and increased surgical site infections (18, 29–31); however, some large studies of hundreds of patients describing the effects of cigarette smoking in spinal surgery have not found smoking to be associated with negative outcomes or complications in the surgical treatment of cervical spondylotic myelopathy, adult deformity surgery, decompression/fusion in degenerative lumbar spinal stenosis, multi-level cervical spine decompression, and anterior cervical spine surgery (32–36). Nevertheless, a recent subset analysis of the SPORT spinal stenosis cohort showed that all patient subgroups with more than 50 patients improved with surgical treatment except for smokers, as smoking was an independent risk factor for not improving with surgical treatment (37). A separate large study with 2-year follow-up of 4,555 patients who underwent surgery for lumbar spinal stenosis, with or without fusion, recently showed that smokers were more likely to be dissatisfied with surgery, used analgesics more regularly, and were less likely to have significant improvement in ambulation (38). An additional study of 203 patients who had lumbar decompression and posterolateral fusion found that smokers had less leg pain relief when compared with their non-smoking counterparts (39). Lastly, a study of 23 patients also showed that, compared with smokers, non-smokers had more postoperative relief of arm pain after anterior cervical decompression and fusion with a specific fusion cage (40).

Given the deleterious effects of smoking on surgical patients, it is not surprising that the benefits of perioperative smoking cessation, including improved surgical outcomes and fewer wound and other postoperative complications, are well-documented in multiple studies and systematic reviews (9, 10, 41–45). Furthermore, one orthopedic study evaluating total knee and hip arthroplasty demonstrated perioperative smoking cessation led to an overall reduction in the cost of care (22). Perioperative smoking cessation is a unique opportunity to reduce complications and the overall cost of healthcare, because surgery is one of the rare occasions where continued smoking may cause immediate negative consequences for the patient, in contrast to the typically delayed consequences of smoking (9, 20). Spinal surgery provides a critical opportunity since studies have shown that patients undergoing major surgery, as opposed to an outpatient procedure, have rates of smoking cessation as high as 35–50%, with some patients continuing smoking cessation even after their recovery (20).

The most significant limitation of this study is the nature of the data collection. The number of patients (>100,000) precludes the data acquisition and subsequent analysis of specific individual spinal pathology characteristics (e.g., radiographic extent of spinal stenosis, symptom type/duration) other than the basic information provided by the diagnostic ICD-9-CM codes, procedure codes, and other easily tabulated patient history (e.g., presence or absence of smoking, obesity). Although these characteristics could have an effect on patient outcome and costs, the large patient numbers, coupled with the fact that there is no reason to believe that the smoking and non-smoking patients were dissimilar enough to skew the results, lends confidence to the results. Obese patients were specifically excluded during the data acquisition so that the pure effects of smoking could be seen without the results being influenced by patient obesity, which is associated with increased cost, complications, and infections in spinal surgery patients (16, 17, 19). The lower smoking rate observed in the patients in this study (10 vs. 30% of all surgical patients) may be explained by the exclusion of obese patients, as smokers have recently been shown to have higher rates of obesity and diabetes, especially in moderate and heavy smokers (46, 47). The low smoker rate in this study may represent another limitation with respect to the generalizability of these initial results.

## Conclusion

Non-obese patients who smoke tobacco and were admitted to UHC hospitals from 2005 through 2011 with spinal dorsopathy, fracture, or congenital pathology had worse outcomes, more complications, and higher total direct costs than their non-smoking counterparts. The smokers in the curvature group also had higher 14- and 30-day readmission rates. This report also documents increased cost associated with smoking tobacco in spine patients; for smokers, the mean total cost for the four most common spine pathologies is $38,697, whereas the same costs for a non-smoker average $32,270 (*p* < 0.001). The Patient Protection and Affordable Care Act’s emphasis on cost control and payment for performance, in addition to the universal goal of providing the best possible outcomes for patients, demands that surgeons advise their potential spine surgery patients to enroll in formal smoking cessation programs prior to consideration of surgery. Further studies are necessary to better define the association between smoking tobacco and worse outcomes in spine surgery patients.

## Conflict of Interest Statement

The authors declare that the research was conducted in the absence of any commercial or financial relationships that could be construed as a potential conflict of interest.

## Appendix

**Table A1:** **Complications per 1,000 patient encounters for spinal disease 2005–2011**.

Complication description	Rate per 1,000
Postoperative pulmonary compromise	38.97
Mechanical complications due to device or implant	27.62
Other complications of procedures	24.61
Aspiration pneumonia	15.96
Venous thrombosis/pulmonary embolism	12.42
Nosocomial pneumonia	15.87
Miscellaneous complications	12.32
Sepsis	10.77
Wound infection	9.90
Post-procedure hemorrhage or hematoma	9.07
Acute myocardial infarction occurring during hospital stay	7.27
Procedure-related perforations or lacerations	6.38
Cellulitis or decubitus ulcer	5.18
Reopening of surgical site	4.47
Postoperative stroke	4.13
Gastrointestinal hemorrhage	4.04
Central or peripheral nervous system	3.85
Postoperative infections not pneumonia/wound	3.28
Catheter-associated urinary tract infection	1.49
Postoperative urinary tract complication	1.17
Postoperative cardiac abnormality except acute myocardial infarction	0.82
Hospital-acquired C. diff enteritis	0.69
Postoperative coma or stupor	0.58
Adverse events due to anesthesia	0.36
Postoperative physical and metabolic derangements	0.23
Readmission for infection due to previous care	0.22
Infection/inflammation due to internal device, implant, graft	0.14
Readmission for other complications of internal device, implant, graft	0.07
Shock or cardiorespiratory arrest	0.03
Post- or intraoperative shock due to anesthesia	0.01

*Rates for complications with rates >1 per 100,000*.

## Abbreviations

ICU, intensive care unit; LOS, length of stay; UHC, University HealthSystem Consortium.
